# Transactions of the Pathological Society of Philadelphia

**Published:** 1840-01-11

**Authors:** 


### MEDICAL EXAMINER.

DEVOTED TO MEDICINE, SURGERY, AND THE COLLATERAL SCIENCES.

No. 2.] PHILADELPHIA, SATURDAY, JANUARY 11, 1840. [Vol. III.

### TRANSACTIONS OF THE PATHOLOGICAL SOCIETY OF PHILADELPHIA.

*December 30th*, 1839.

The President, Dr. Gerhard, in the Chair.

*Case of Rheumatism, Endo-pericarditis, Pleurisy, and Double Pneumonia, with Autopsy.* By Dr. Stillé.

I. L., æt. twenty-three, a seaman, had always enjoyed good health, and was naturally of a vigorous constitution. He had just returned, in good health, from a two years' cruise, when, on November 30th, he was exposed to a cold rain, after leaving a warm room, and on his way to one less comfortable. Within an hour after this exposure, he was attacked by a severe pain over the cartilages of the left false ribs, so acute and violent as to prevent him from drawing his breath freely, and from sitting in an upright position. The pain lasted from one to two hours,—and then, after the administration of some warm drinks, passed off. On the 1st and 2d of December he had no pain, nor dyspnœa, nor did he feel otherwise unwell; but his appetite was less than usual. On the 3d inst. the pain returned in the same place, without any known exciting cause, with less violence than before, but was aggravated by any movement of the body, and by forced inspiration. He had no headach, heat of skin, nor unusual thirst, and neither nausea nor diarrhœa. On the 4th December, he entered the Pennsylvania Hospital. I found him complaining of pain about the cartilages of the seventh and eighth false ribs, and in a space extending for two or three inches from them in every direction, increased by moderate pressure, and by full respiration; no cough, nor expectoration; the patient was sitting on his bed-side; said he preferred lying on his right side, but had no pain when on his left side. The respiration was every where soft and vesicular, without marked expiration. Percussion was every where resonant behind; it was not carefully made before. The sounds of the heart were distinct and natural.

Eight ounces of blood were taken by cups from the seat of pain, affording marked relief, which was still further increased by the application of a blister. Several doses of laxative medicine were given in the first three days, and the wine of colchicum prescribed in the dose of twenty drops three times a day. All the symptoms abated; and the patient continued to walk about the ward until the night of the 8th December, when he was seized with a pain, acute and violent, like the previous ones, but situated directly over the heart, and extending thence to the whole left side of the trunk. The dyspnœa was extreme. A sinapised foot-bath was ordered, and a mustard poultice to the præcordium. The pain diminished,—but, several hours afterwards, the dyspnœa persisted, accompanied with an anxious and pale face, and a livid circle around the eyes; decubitus on the left side. I then took sixteen ounces of blood from the arm. The pain and dyspnœa were at once relieved, and continued less during the 9th; on the evening of that day I prescribed an anodyne mixture. L. passed a comfortable night. On the 10th, I examined the region of the heart, and found dulness on percussion, with a marked convexity of the chest, extending from the second rib down to the seventh, and from the right side of the sternum to the left nipple. There was no visible nor sensible impulse of the heart; and its sounds were very dull, and as if remote. Eight ounces of blood were taken by leeches from the præcordial region; before this quantity of blood had been withdrawn, the patient was able to lie upon his back, and breathe easily in that position.

In the evening he said he felt like "a new man." A poultice was then applied over the leech bites, and ten grains of calomel, and as many of rhubarb, prescribed.

11*th.*—Had slept half the night; two stools; continues to feel much better; decubitus dorsal; face rather sallow and thin; expression less anxious; pulse 90, regular, small; respiration 30; præcordial dulness rather less, extending only to middle line of sternum; its other limits as before; the sounds dull, but their other characters natural. (℞. Blister over heart.) *Evening.*—Pulse 108; respiration 40. From the 11th to the 16th, L.'s condition did not change materially; his respiration was rather freer, and the pain very slight. He could not sleep at night. On the 14th he took ten grains of lupulin, without sleeping; on the 15th he took half a grain of sulph. morphia, and slept about seven hours.

16*th.*—Decubitus dorsal; face sallow, except the cheeks, which have a hectic flush; expression very anxious; no headach; intelligence and senses perfect; pulse 104, small and quick; respiration 44, laborious and painful; tongue dry, centre coated with a rough brown crust; he breathes through his mouth; no nausea nor vomiting; thirst considerable, avoids cold drinks; anorexia; two stools after purgative enema; abdomen soft, not tympanitic; skin warm, harsh, and dry. The left side moves but little in inspiration; there is evident prominence of all the præcordial region; with every breath there is an acute pain half an inch within the left nipple; respiration vesicular, but shrill on right side of chest, before; on left side, there is feeble inspiration and blowing expiration under the clavicle. Percussion resonant on the right side; on the left it is obscure, or flat, from the nipple to the right edge of sternum, and from the lower edge of the sixth rib to the upper edge of the second. Respiration is faintly heard from the second to the fourth rib. There is no sensible impulse of heart; the sounds of this organ are dull and distant, their rythm is natural. Diet: gruel, barley and gum water. Six ounces of blood to be taken by leeches from over the heart. The relief afforded by this application was prompt and decided.

*17th.*—Pulse 128. Respiration 40. No sleep last night; head warm; expression anxious; features haggard; skin of face greasy; circumscribed flush on cheek; nostrils expand in breathing; great prostration. Same signs offered by thorax, except that under the middle third of the left clavicle there is metallic resonance on percussion, with amphoric respiration, and pectoriloquy. Below the innermost third of this clavicle there is perfect flatness on percussion, with bronchial voice and respiration. ℞. Calomel gr. j.; opium gr. 1-6th every two hours. Poultice over heart, and to legs.

*Evening.*—General symptoms as in the morning. Pain at the junction of sixth left rib with the sternum; expression very anxious; great dyspnœa; pulse 120, quick, of moderate force. Patient sat up while 12 oz. of blood were taken from his arm. Posterior part of chest examined: on left side, there was absolute flatness on percussion from the summit to the base of the lung, and from the spine to the middle of the flank. Respiration was very feeble in all this space; there was bronchial respiration and voice in the upper third, and indistinct œgophony at the root of the lung; in the lowest third respiration was almost extinct. Anterior to the vertical from the axilla, percussion was resonant. On the right side, respiration was puerile in the upper half, and rather feeble in the lowest third. Percussion on this side resonant. Patient prefers lying on his back, or on his left side, but can lie on his right side without great inconvenience. ℞. Blister to left side, behind.

*18th.*—Same general symptoms, except that the respiration is freer; slept none last night; the blister drew well; at the upper part of the left lung, where the sonoriety was so marked, there is now perfect flatness on percussion; the only portion of the left side of the chest at all resonant, is a space of about two square inches above and exterior to the nipple. The amphoric respiration above the third rib very strong, and the vibration of the voice metallic. Decubitus on right side impracticable. No cough. Pulse 112, of moderate force and volume. ℞. Ext. Hyosciam. gr. v., every hour of evening till sleep is procured. Calomel and opium continued.

*19th.*—Slept for about four hours, at intervals; felt refreshed; had profuse sweat during the night; expression less anxious; face less flushed; tongue moister; præcordial prominence less; sounds of heart remote and dull; flatness on percussion extends from the left flank to about half an inch to the right of the sternum, and from the sixth rib to the clavicle; in all this space, except immediately over the heart, there is blowing respiration and vocal resonance, but no rhonchus, nor respiratory murmur. There is no fossa, either above or below the left clavicle. Pulse 116.

*20th.*—General symptoms rather improved. The prominence over the heart less marked; the sounds of the heart more audible; the left side of the chest moves rather more than it did; supra-clavicular space less prominent; at the junction of the second and third ribs with the sternum, there is a little resonance on percussion. Respiration as before.

*22d.*—Same general symptoms. Pulse 100. Respiration 36. Gums slightly ulcerated; a faint mercurial odour in breath; left side of chest regularly rounded; no intercostal depressions. Between the third and fourth ribs the sounds of the heart are distinctly heard, and the ear receives a perceptible impulse at that point. No anormal sound.

*23d.*—Pulse 112, quick, more feeble. Respiration 32, regular; the inspiration is quick and jerking. Expression still anxious; the skin of the face moist and greasy; cheeks flushed; mouth half open; nostrils dilated; teeth brownish; gums more ulcerated; mercurial fœtor marked; the skin of the trunk and extremities moist and warm. Left side of chest, anteriorly, flat on percussion as before; no respiration heard in it; only a faint sonorous rhonchus in the axilla. Over the right lung there are heard sibilant and sonorous rhonchi. Full inspiration causes no pain. Debility more marked. Anorexia; thirst very great. Omit calomel and opium; cataplasm to left side.

*24th.*—Pulse 104, feeble, small, not so quick. Respiration 36, difficult and painful. Slept only an hour last night; was very restless. Complains of pain near left nipple. Sounds of heart feebler, and its impulse less. No cough. Chicken soup, and arrowroot.

*25th.*—Between 5 and 6 o'clock, A. M., was called to see L. Found him in great difficulty of breathing; a loud rattle in the trachea. Face expressive of intense anxiety; hands cool; pulse scarcely perceptible at wrist; pupils dilated; skin covered with profuse and greasy sweat. Sinapisms were applied to the epigastrium and nuchæ; the thorax rubbed with hot whiskey; and warm drinks, with wine, administered by the mouth. He gradually reacted. At 10, A. M., the rattle had ceased, the pulse returned, the expression was more natural. A teaspoonful of brandy was ordered to be given every twenty minutes, and ten drops of the spts. of turpentine, in emulsion, every two hours. At 6 P. M., there was no change for the worse, and the treatment was continued. During the night, symptoms of suffocation again appeared, and were relieved by the same means as before.

*26th.*—Pulse 112, of moderate force. Respiration 36, spasmodic, laborious, and accompanied by groans; slept a little last night; sweated profusely; face thinner, and of a leaden hue; expression very anxious; nostrils dilated, and lower lip drawn down in inspiration; intelligence and senses perfect; deglutition easy; no vomiting; cough rare, and slight; no expectoration; decubitus on the left side; percussion over right lung, before and behind, resonant in upper three-fourths, and obscure in the lowest fourth,—in this latter space there is a fine crepitant rhonchus. Placed in a sitting posture on the side of the bed, L. sustains himself well; percussion over whole of left side of chest, behind, is flat; under the left clavicle it is tympanitic. Respiration faintly heard in upper half of left side of chest behind; doubtful, if audible, in lower half.

*27th.*—Respiration 44, gasping, laborious, quick; expression very anxious; face haggard, and of a leaden hue; pupils natural; eyes dull; profuse perspiration. Pulse 136, very feeble and quick; some delirium; deglutition difficult; decubitus on left side. Over lower half of right side there is dulness on percussion, with crepitant rhonchus in inspiration, and mucous and sonorous rhonchus in expiration. The latter sounds are also heard in the upper half of this lung.

About 2 P. M., after taking some gruel, L. was seized with symptoms of suffocation, and died in about ten minutes.

*Autopsy; twenty-four hours after death.*

*External appearance.*—Emaciation and rigidity moderate; the skin pale throughout, somewhat sallow on the face. Percussion was flat over the whole anterior portion of the left side of the chest, and as far as the right edge of the sternum. The right side of the chest was resonant from the clavicle down to the fourth rib, below which there was dulness on percussion which amounted to positive flatness on the right flank. In this latter region the dulness was considerable, even as high up as the axilla. The form of the left side of the thorax was evidently more rounded, and its prominence was greater than on the opposite side, the intercostal spaces being more clearly defined on the latter side.

*Dissection.*—The muscles were firm, and of a bright red colour. *The right lung* accurately filled its cavity, and its pleura was connected with that of the ribs by cellular adhesions of considerable firmness, but not strong enough to resist the pressure of the fingers. The lowest lobe of this lung was dense, it contained no air, and sank in water; its tissue was of a grayish red colour, offering on section, a granulated surface, and was easily reducible to a pulpy mass by moderate compression. The middle lobe offered nearly the same characters, but its consistence was rather greater, and its colour was not so pale. The upper lobe yielded on incision, a considerable quantity of a brownish, frothy liquid; it was of a redder colour, and firmer consistence, especially its anterior portion; a piece cut off from this latter part, floated upon water. The bronchiæ were red, and their lining membrane thickened and softened.

*Left lung.*—In the upper half of the pleural cavity there was a pint and a half of turbid liquid which, when removed, disclosed a corresponding cavity, lined by a false membrane, of a yellow colour, from one to two lines in thickness, very firm, and having on its free surface, flakes of lymph only partially attached. This membrane was easily removable in small portions, and beneath it the pleura was found smooth and shining, but injected. The hand introduced into the cavity, discovered it to be of a regular form above, corresponding to that of the upper part of the chest, but communicating, by several prolongations, with smaller cavities towards the base of the lung. The lung itself was firmly fixed by the false membrane in the lower and inner corner of the left side of the thorax, bound against the spine, and the tendon of the diaphragm, so that it could not be removed without laceration.

The tissue of the lower lobe had a fleshy aspect, and was, in general, of a reddish gray colour, but in some spots the red, and in others the gray tinge, predominated, which, with the granular state of its section, gave it an appearance somewhat like that of the cut surface of a kidney, divided parallel to its long axis. Its density was sufficient to cause it to sink in water, and its consistence, although greater than that of the lowest lobe of the right lung, was inadequate to resist firm pressure. The upper lobe of this lung was of a pretty uniform, grayish colour, more supple and more firm than the lower lobe, but contained no air whatever. The bronchiæ of this lung, like those of the right, and the trachea, had their lining membrane of a vivid red, and somewhat softened. There were no tubercles in either lung.

*Heart.*—The cavity of the pericardium contained about half a pint of yellowish, semi-transparent fluid; a reddish-white false membrane completely lined the cavity, and there were bands of it connecting the opposite surfaces in many points. This false membrane had a reticulated or cellular disposition, on the internal face of the pericardium, while on the heart it was smoother, and more compact, and varied between one and two lines in thickness. The substance of the heart was pale and soft; the volume of the whole organ, and the thickness of its muscular walls, offered no marked deviation from the ordinary condition. The right auricle contained a large and very firm coagulum, of a pale colour, intimately connected by one extremity with the folds of the tricuspid valve, its substance being of a reddish-brown hue, and of a fibrous texture, and its surface covered by a perfect membrane, resembling, in its transparency, and other physical characters, a true serous membrane. A similar but smaller coagulum existed in the right ventricle. The surface of this latter cavity presented numerous patches of a pearly white colour, through which the redness of the muscle shone faintly. The folds of the tricuspid valves were wrinkled, their free edges of a bright red hue, and their surfaces marked by superficial and thin milk-white patches, and by little circumscribed elevations of the same colour, but more opaque. The same characters belonged to the lining membrane of the left side of the heart, and the mitral valve. The semilunar valves of the aorta were less supple than natural, and were either red, or covered by white patches. The lining membrane of this artery was of a vivid red colour, and could easily be raised from the middle coat of the vessel, which preserved its natural colour. The valves of the pulmonary artery were supple, but not quite as transparent as natural; the lining membrane of the vessel was injected like that of the aorta, but in a less degree.

*Recapitulation.*—A seaman, æt 23, of good constitution, is transiently exposed to cold and wet, and is immediately afterwards seized with a pain over the left false ribs, which lasts for an hour or two and then leaves him to return again on the fourth day; he has no cough, nor expectoration, nor do the physical signs afforded by the thoracic organs show them to be affected. On the eighth day the patient is taken with severe pain over the heart, and great dyspnœa, both relieved by venesection. On the tenth day there are evident signs of an effusion into the pericardium, of which the general symptoms very much diminish, and the physical signs show some amelioration during the following four days. But on the next succeeding day the 16th, namely, from the commencement of the attack, there arise new signs of disorder in the circulation and respiration; over the summit of the left lung, anteriorly, bronchial respiration is heard, and percussion is dull in the same region. On the following day percussion is tympanitic under the middle of the left clavicle, where dulness existed the day before, and is accompanied by pectoriloquy. Behind, there is absolute flatness on percussion, from the summit to the base of the left lung; feeble respiration in the lowest third; œgophony at the root of the lung, and broncophony in its superior third. On the next day, (the 18th,) percussion is again dull under the left clavicle, but the characters of the respiration, and the voice are there the same as before. The 19th, the left clavicular fossæ are observed to be filled up. The 20th, there is some subsidence of the præcordial prominence. The 23d, the sounds of respiration are no longer heard under the left clavicle; those of the heart have become more audible. On the 25th the respiration becomes very difficult; and, the 26th, crepitant rhonchus and dulness on percussion are observed in the lower third of the right lung, hitherto uninvolved, and the tympanitic resonance is again found under the left clavicle.

On the 27th the patient dies. At the examination of his body, the pericardium is found to contain a quantity of serum, filling, without distending it. Its cavity is lined by a recent false membrane, and the interior of the heart presents signs of inflammation, consisting in a milky appearance of certain portions of its lining membrane, a vivid redness of other portions, including the internal coat of the aorta, and the formation of organized coagula. There is pneumonia of the inferior lobes of both lungs, and solidification of the upper lobe of the left, from the compression of a false membrane, lining the whole pleural cavity of that side, and a serous effusion, distending the upper half of the same cavity, so that no portion of the left lung rises above the level of the third rib. There are no tubercles in either lung.

*Remarks.*—It might at first excite surprise that such extensive organic changes should be found twenty-seven days after the first appreciable alteration of the patient's health, and nineteen only from the first symptoms of serious disorder of his functions, and some suspicion might be indulged that the case was not examined with sufficient care on its first coming under observation. But the record, although imperfect, is full enough to establish the negative fact, that no inflammation of the thoracic viscera existed during the first seven days. The only affection that revealed itself during this period, was pain over the left false ribs, which, from its mode of origin, its seat, and the want of any sympathy shown by the system, was considered rheumatic. That pericarditis should succeed rheumatism, is in accordance with abundant experience, but as the latter disease does not commonly affect the cartilages of the ribs alone, it must be admitted that, in the present case, the rheumatic nature of the pain over those bodies admits of some doubt, especially as, in the course of time, a neighbouring organ became the seat of violent morbid action. This organ may have been disordered from the beginning, and have caused pain where it was actually felt; but on the other hand, it is certain that when the pericardium became unquestionably inflamed, the pain was referred to its usual seat under such circumstances, over the heart, and not to the false ribs. We think, then, that the patient was first attacked by rheumatism of the left false ribs because of the seat of pain, because the constitution showed but little interest in the disease, because of the effects of the treatment, because the pericardium gave no signs of inflammation at first, and did give such signs afterwards; and, finally, because when the pericarditis had evidently begun, the pain changed its seat, and showed itself near the affected organ.

After the heart, the left lung was attacked by pneumonia, and pleurisy, with effusion. Without enlarging upon this interesting succession of events, we cannot refrain from noticing a curious physical phenomena. On the 16th day there was found, under the left clavicle, *dulness on percussion*, with bronchial respiration and resonance of the voice; on the following day, under the middle third of the same clavicle, the same phenomena were found on auscultation, with *tympanitic resonance on percussion*; on the succeeding day, percussion over the same part was again *dull*; and, ten days afterwards, the percussion was once again *tympanitic*; the following day, the left cavity of the pleura was found distended by fluid, the lung bound down to the spine, and no portion of it within several inches of the spot where percussion had given such varying results. An attempt to explain these phenomena by supposing, according to Williams, that the trachea caused the reverberation of sound, is at once shown to be fruitless, from the fact that the resonance was under the middle third of the clavicle, and that dulness existed, in each instance, under the inner third of that bone, under the part, namely, in contiguity with the trachea. We might make several hypotheses as to the cause of these singular physical signs, but as none of them have appeared satisfactory to our own mind, we refer the matter to others who may have met with similar anomalous cases, and have either had a better opportunity of explaining them, or have availed themselves of it more thoroughly than ourselves.

We are shown by the history of this case, that diseases, apparently unimportant in themselves, are not, on that account, to be slightly examined, or treated carelessly. We should always keep in view, not only the affection itself, but also its tendencies; remembering "how great a flame a little fire kindleth." In this instance a slight attack of a disease more regarded commonly for the transient pain it inflicts, than for the permanent injury it entails, was the first small link in a chain, which at last fettered and destroyed a young and vigorous life.

